# The role of melatonin in the development of postmenopausal osteoporosis

**DOI:** 10.3389/fphar.2022.975181

**Published:** 2022-10-07

**Authors:** Keda Yang, Xueshan Qiu, Lili Cao, Shui Qiu

**Affiliations:** ^1^ Department of Orthopedics, First Hospital of China Medical University, Shenyang, China; ^2^ Department of Pathology, The First Affiliated Hospital of China Medical University and College of Basic Medical Sciences Shenyang, Shenyang, Liaoning, China; ^3^ Department of Medical Oncology, First Hospital of China Medical University, Shenyang, China

**Keywords:** melatonin, postmenopausal osteoporosis, biological rhythms, antioxidant, anti-inflammation, immuomodulation, gut microbiota

## Abstract

Melatonin is an important endogenous hormone that modulates homeostasis in the microenvironment. Recent studies have indicated that serum melatonin levels are closely associated with the occurrence and development of osteoporosis in postmenopausal women. Exogenous melatonin could also improve bone mass and increase skeletal strength. To determine the underlying mechanisms of melatonin in the prevention and treatment of postmenopausal osteoporosis, we performed this review to analyze the role of melatonin in bone metabolism according to its physiological functions. Serum melatonin is related to bone mass, the measurement of which is a potential method for the diagnosis of osteoporosis. Melatonin has a direct effect on bone remodeling by promoting osteogenesis and suppressing osteoclastogenesis. Melatonin also regulates the biological rhythm of bone tissue, which benefits its osteogenic effect. Additionally, melatonin participates in the modulation of the bone microenvironment. Melatonin attenuates the damage induced by oxidative stress and inflammation on osteoblasts and prevents osteolysis from reactive oxygen species and inflammatory factors. As an alternative drug for osteoporosis, melatonin can improve the gut ecology, remodel microbiota composition, regulate substance absorption and maintain metabolic balance, all of which are beneficial to the health of bone structure. In conclusion, our review systematically demonstrates the effects of melatonin on bone metabolism. Based on the evidence in this review, melatonin will play a more important role in the diagnosis, prevention and treatment of postmenopausal osteoporosis.

## 1 Introduction

Postmenopausal osteoporosis is a common disease of bone metabolism occurring in women after amenorrhea (Farlay et al., 2022). The onset of osteoporosis is insidious without specific symptoms. It is usually diagnosed after the occurrence of serious complications, including pain, spinal deformity and fracture (Chow et al., 1989). Osteoporotic fracture is the main factor threatening the health of patients with osteoporosis. Previous studies have indicated that 20% of women suffer from osteoporosis and 10% of these women have fractures in different sites (Wang et al., 2021). Early diagnosis and treatment are essential to prevent osteoporosis complications. At present, dual energy X-ray absorptiometry (DXA) is the gold standard for the detection of bone mineral density. However, DXA test results have obvious limitations for the diagnosis and treatment of osteoporosis. In terms of localization, DXA generally detects the bone density of the waist and hip, but it cannot determine the distribution and heterogeneity of the bone (Silva et al., 2013; Cataño Jimenez et al., 2020). Additionally, DXA is unable to detect the trabecular bone microstructure and predict the risk of fractures (Nazarian et al., 2009). More importantly, DXA testing equipment is expensive, and it is difficult to apply to the whole population, resulting in the omission of patient screening. The development of complementary assays to assess systemic bone mass and fracture risk is essential.

Postmenopausal women are the main high-risk group for osteoporosis (Liu et al., 2022a). The core pathogenesis of postmenopausal osteoporosis is estrogen deficiency. Hormone replacement therapy has been applied in the treatment of osteoporosis. However, exogenous hormones disrupt endocrine homeostasis and increase the risk of breast cancer, endometrial cancer and cholelithiasis (Zhang et al., 2021a). Drug therapy for osteoporosis is divided into two types: promotion of osteogenesis and inhibition of osteoclasts, with the latter being the mainstay (Zhou et al., 2020a). Due to the limitations of detection methods and the lack of awareness during a physical examination, patients with osteoporosis often undergo drug intervention after serious complications occur. Drugs for inhibiting osteoclasts only prevent further bone loss but do not fundamentally improve bone mass. Osteogenesis drugs such as teriparatide can cause endocrine disturbances, gastrointestinal irritation and central nervous system lesions. The development of mild osteogenic drugs is an effective measure to address the limitations of current drug treatments for osteoporosis. In addition, revealing the pathophysiological changes induced by estrogen decline will contribute to determining the pathogenesis and clinical treatment of postmenopausal osteoporosis.

Melatonin is a hormone secreted by the pineal gland. Its functions include adjusting biological clock rhythm, eliminating free radicals, delaying aging and enhancing immunity (Zhang et al., 2020; Guo et al., 2021a; Li et al., 2021a; Zhang et al., 2021b). Melatonin is used as a component of nutraceuticals to maintain body health due to its limited side effects. At present, melatonin is mainly applied to people with sleep disorders and insomnia (da Silveira Cruz-Machado et al., 2021). Melatonin effectively shortened the time to fall asleep, reduced the time of light sleep, increased the time of deep sleep, improved sleep quality, and helped people stay awake after waking up. Additionally, there is good evidence that melatonin can be used to treat ischemia-reperfusion injury, primary headache, and fibromyalgia and can control blood sugar and blood pressure (Leelaviwat et al., 2022). Melatonin is increasingly valued by patients and clinicians due to its antioxidant and antiaging properties. In our previous study, we determined that serum melatonin levels were obviously decreased in postmenopausal women with osteoporosis (Cao et al., 2022). Changes in serum melatonin were significantly correlated with bone metabolism markers in the development of postmenopausal osteoporosis (Ostrowska et al., 2001a). This evidence indicated the potential role of melatonin in the evaluation of bone mass and strength. Additionally, our previous studies also revealed the positive effect of melatonin on osteoblasts and its therapeutic effect in postmenopausal osteoporosis in animals (Da et al., 2020; Wen et al., 2020). Oral administration of melatonin could increase the serum level of melatonin to improve bone mass (Guan et al., 2022). Melatonin decreased the relative ratio of serum osteoclasts and osteoblasts to improve bone balance (Kotlarczyk et al., 2012; Maria et al., 2018). It is obvious that melatonin plays an important role in bone metabolism. In addition to the direct effect on bone cells, some studies also indicated that melatonin could regulate bone homeostasis in different indirect ways (Xu et al., 2018; Zhou et al., 2019). Melatonin is a mild osteogenic drug with few complications and has the potential and prospect of being an effective drug for the treatment of osteoporosis. Clarification of the role of melatonin in resisting the pathological changes caused by estrogen deficiency and the optimization of the drug formulation of melatonin for the treatment of osteoporosis are promising research directions. Therefore, we aim to review the studies that investigated the role of melatonin in the development of postmenopausal osteoporosis, elucidate the mechanism by which melatonin improves bone metabolism, and provide insights into the application of melatonin in osteoporosis treatment.

## 2 Methods

### 2.1 Search strategy

The PubMed, Web of Science, Ei Compendex and Wiley databases were used to search the research literature on melatonin and postmenopausal osteoporosis. We searched any identified studies including reviews, articles, early access, editorial materials, and letters. The results comprised papers available from the inception of the database to July 2022. Search terms included Osteoblast, Osteoclast, Melatonin, Osteogenesis, Osteogenic differentiation, Osteoclast differentiation, Biological rhythms, Antioxidant effect, Anti-inflammation, Immuomodulation, Gut, Gut microbiota, and Postmenopausal osteoporosis. The abstract or full text of these studies was reviewed to check whether they matched the corresponding section.

### 2.2 Melatonin promotes osteoblast differentiation and inhibits osteoclast activity

#### 2.2.1 Molecular mechanism and signaling pathway *in vitro*

**FIGURE 1:**
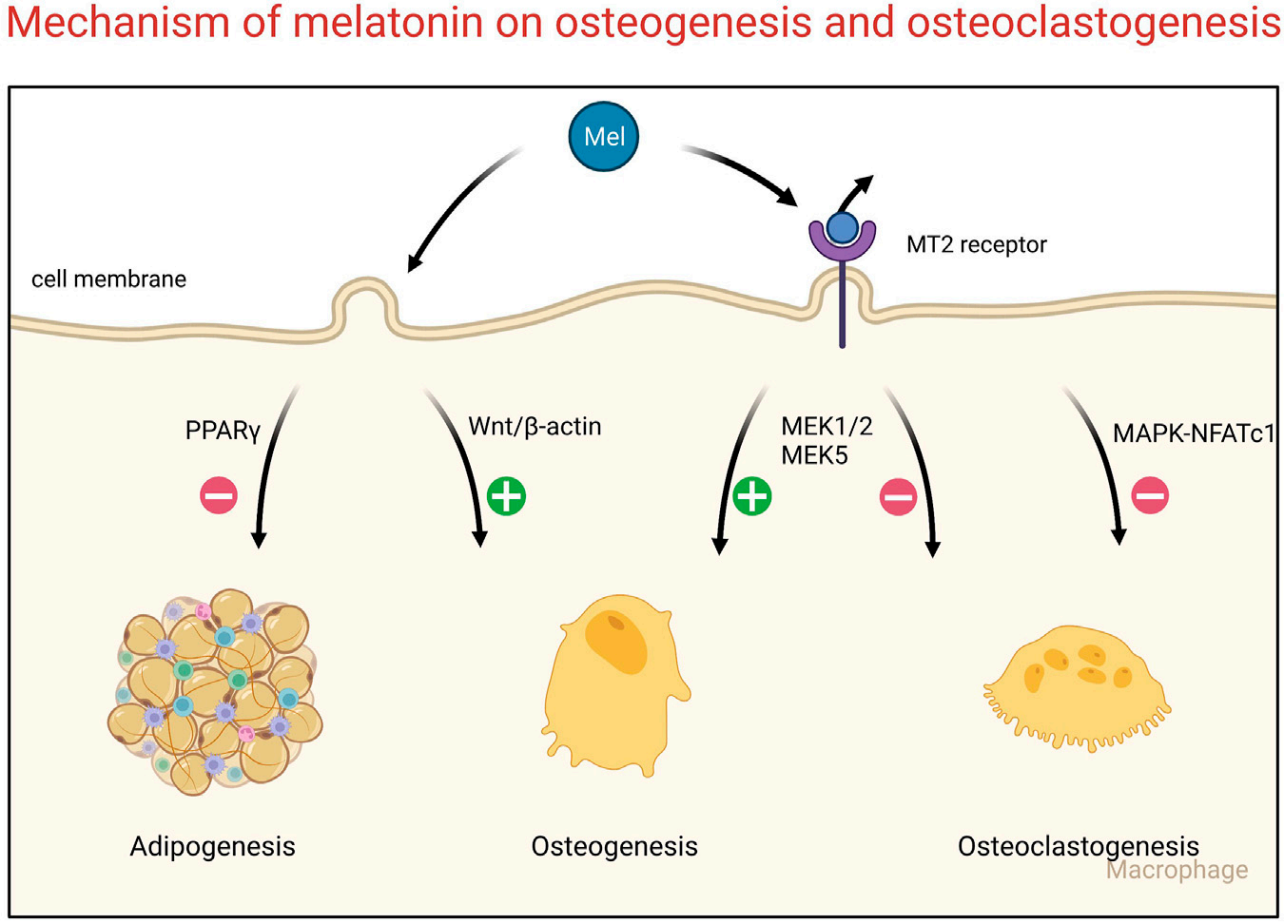
Signaling pathways of bone remodeling regulated by melatonin.

Osteoblasts differentiate from the mesenchymal stem cells in bone marrow (BMSCs) (Sun et al., 2022). Previous studies have indicated that there are two opposite trends of BMSC differentiation that result from the balance between osteogenesis and adipogenesis (Wang et al., 2022a; Suo et al., 2022). Melatonin could increase the expression of osteogenic markers of mesenchymal stem cells and contribute to the mineralization of the bone matrix (Guan et al., 2022). Melatonin promotes the osteogenic differentiation of BMSCs *via* the Wnt/β-catenin pathway and inhibits the adipogenic differentiation of BMSCs *via* the PPARγ pathway (Zhang et al., 2010; Han et al., 2021). In pathological conditions, melatonin could also resist cell damage and prevent osteoblast function (Zhao et al., 2020; Gong et al., 2022). As mentioned above, melatonin is a potential osteogenic promoter in bone metabolism. Melatonin combined with the MT2 receptor could induce signaling transduction in osteogenesis and promote ossification (Zhang et al., 2021c). However, MT2 activation also suppresses osteoclastogenesis activity by inactivating the NF-kappaB pathway (Zhou et al., 2020b). In coculture conditions, exogenous melatonin promotes the osteogenesis of mesenchymal stem cells and inhibits the osteoclastogenesis of peripheral blood monocytes *via* the MT2-mediated MEK1/2 and MEK5 pathways (Maria et al., 2018). Additionally, melatonin decreased the expression of RANKL and further suppressed the activity of osteoclasts *via* the receptor-independent MARK and NFATc1 pathway (Kim et al., 2017; Kim et al., 2022) (Figure 1).

#### 2.2.2 *In vivo* experiments

Melatonin also modulated the estrogen receptor on osteoclasts to maintain the inhibitory effect on osteoclast differentiation (Suzuki and Hattori, 2002). In vivo experiments, estrogen-deficient mice were obtained by bilateral ovariectomy (OVX). Melatonin feeding could improve bone mass and relieve bone loss in OVX mice (Gürler et al., 2019). Histological detection of bone tissues indicated that melatonin increases the expression of Collagen I and BMP2 but decreases the expression of PRMT1 and TRAP (Choi et al., 2021; Huang et al., 2021). These data demonstrate that melatonin has positive effects on osteogenesis and negative effects on osteoclastogenesis in animal models, which is consistent with the results at the cellular level. In conclusion, melatonin plays an important role in bone balance through direct regulation.

The direct mechanism of melatonin in regulating bone metabolism is summarized and listed in Table 1.

**TABLE 1:** The mechanism of melatonin regulating bone remodeling.

Biological function	Targets and signaling pathways	Citation
Promotion of osteogenesis	Wnt/β-catenin pathway, MT2 receptor	Han et al. (2021), Zhang et al. (2021c)
Suppression of adipogeneis	PPARγ pathway	Zhang et al. (2010)
Suppression of osteoclastogenesis	NF-kappaB pathway, MT2 receptor, MEK1/2 and MEK5 pathway, MARK and NFATc1 pathway	Zhou et al. (2020b), Maria et al. (2018), Kim et al. (2022), Kim et al. (2017)
Increase of osteoblast markers	Collagen I and BMP2	Huang et al. (2021)
Decrease of osteoclast markers	PRMT1 and TRAP	Choi et al. (2021)

a Osteoporosis is a common metabolic disease of bone tissue characterized by bone resorption of osteoclasts over bone formation of osteoblasts. MT2 receptor, melatonin receptor 2; PPARγ, peroxisome proliferators-activated receptor gamma; NF-kappaB, nuclear transcription factor-kappaB; MARK, mitogen-activated protein kinase; NFATc1, Nuclear factor of activated T cells 1; BMP2, Bone morphogenetic protein 2*;* PRMT1, protein arginine methyltransferase 1; TRAP, triiodothyronine receptor auxiliary protein.

### 2.3 Biological rhythms

Biorhythm plays an important role in the development of postmenopausal osteoporosis. A recent study showed that the endochondral bone formation process exhibits a biological rhythm characterized by rapid DNA replication and active cellular mitosis during the day, followed by matrix synthesis at night (Yu et al., 2022). The circadian clock regulates the biological cycle of mammalian physiological functions. Biological regulation maintains energy metabolism in bone tissue, including the metabolism of glucose, amino acid and fat (Luo et al., 2021).

#### 2.3.1 Clinical data

Bone metabolism markers were secreted in a special circadian rhythm (Zheng et al., 2021). Rhythmic regulation of bone metabolism hormones, such as parathyroid hormone and growth hormone, maintains the homeostasis of calcium, phosphate, Collagen I peptide and 1,25-dihydroxyvitamin D in bone tissue (Joseph et al., 2008). Circadian clock disturbance led to the disorder of bone metabolism when clock-related genes were knocked out or sleep restriction occurred (Song et al., 2018). As the circadian clock was disturbed in postmenopausal women, the process of bone formation, energy metabolism and the expression of turnover markers in bone tissue, as well as the secretion of bone metabolic hormones were disordered (Kruijver and Swaab, 2002). Melatonin is a type of rhythmic hormone. Its secretion increases with weak light stimulation at night but decreases in the daytime (Carstensen et al., 2022). The rhythmic regulation of melatonin affected the metabolism of nutrients, including carbohydrates, lipids and proteins, indicating the important role of melatonin in maintaining body functions (Kent et al., 2022; Qian et al., 2022). A previous clinical study demonstrated the protective effect of melatonin in rhythmic secretion on bone metabolism in postmenopausal women (Ostrowska et al., 2001b).

#### 2.3.2 Modulatory mechanism of melatonin in the biological rhythm of bone metabolism

The circadian levels of Collagen I biomarkers were suppressed when melatonin concentrations were altered during the day in postmenopausal women. In addition, serum carboxyterminal telopeptide of type I collagen (ICTP) together with urinary hydroxyproline and Ca was negatively correlated with the serum melatonin level (Ostrowska et al., 2001a). Melatonin also improved bone mass in a rhythmic regulatory method by exogenous supplementation in addition to its relationship in physiological and pathological conditions. Circadian clock genes were closely associated with bone metabolism (Table 2). CLOCK gene mutations inhibit the combination of 1,2,5(OH)_2_D_3_ and the PDIA3 receptor, which is involved in bone remodeling (Yuan et al., 2017). Melatonin increased the expression of CLOCK (Wan et al., 2020). As the downstream target of CLOCK, BMAL1 also led to the occurrence of osteoporosis when the factor was defective (Chen et al., 2020a). Inactivation of BMAL1 suppressed the differentiation of osteoblasts and enhanced the function of osteoclasts. Melatonin induced AMPKβ1 phosphorylation to increase BMAL1 expression by activating melatonin receptor 1 (Yu et al., 2022). REV-ERB/ROR is a group of response elements that modulate the expression of BMAL1 (Lee et al., 2016). Melatonin suppressed osteoclastogenesis by increasing the expression of REV-ERB (Tian et al., 2021). As mentioned above, the melatonin level is closely related to the expression of bone metabolism biomarkers. Disruptions in the circadian rhythm of melatonin secretion lead to an imbalance in bone remodeling, and exogenous melatonin plays a positive role in bone homeostasis by modulating circadian clock genes and factors.

**TABLE 2:** Circadian clock genes and mechanism regulated by melatonin in bone metabolism.

Circadian clock genes	Mechanism in regulating bone metabolism	Citation
CLOCK	Activating the PDIA3 receptor of 1,2,5(OH)_2_D_3_	Yuan et al. (2017)
BMAL1	Promoting osteogenesis and suppressing osteoclastogenesis	Chen et al. (2020a)
REV-ERB/ROR	Suppressing osteoclastogenesis	Tian et al. (2021)

a Melatonin is a circadian rhythm-regulating hormone secreted by pineal gland. BMAL1, Basic helix-loop-helix ARNT, like 1; PDIA3, protein disulfide isomerase family A member 3.

### 2.4 Antioxidant effect

Estrogen is a type of antioxidant hormone that regulates the function of mitochondria to maintain the activity of oxidase and the production of oxidants (Miyazaki-Akita et al., 2007; Yung et al., 2011). Disturbances in the antioxidant systems lead to imbalances in bone metabolism (Yang et al., 2022a). Postmenopausal osteoporosis is a disease caused by oxidation-reduction disorders due to estrogen deficiency (Yang et al., 2021). On the one hand, oxidative stress weakens the function of osteogenesis (Lian et al., 2021). On the other hand, reactive oxygen species (ROS) signals could activate RANKL-mediated osteoclastogenesis (Li et al., 2022). Both effects resulted in bone mass loss in bone tissue.

#### 2.4.1 Effect and molecular mechanism

Melatonin is the most powerful endogenous free radical scavenger. Its main antioxidant mechanism acts through its binding to oxidative substances (Lu et al., 2022). Additionally, melatonin binds to its cellular receptor and activates the transduction signals that induce the synthesis of antioxidant enzymes, including superoxide dismutase, catalase, glutathione peroxidase, and glutathione reductase (Bantounou et al., 2022). To prevent oxidative damage, melatonin suppresses the cellular senescence induced by oxidative stress in bone marrow mesenchymal stem cells to maintain the osteogenic differentiation (Chen et al., 2022). Melatonin also increases the expression of SIRT1, which is a deacetylase that is closely involved in mitochondrial biosynthesis to improve oxidative damage in osteoblasts (Chen et al., 2020b; Liu et al., 2022b). In addition, melatonin promotes SIRT3-mediated antioxidase production to hydrolyze free radicals and thereby protect osteoblasts against apoptosis (Zhou et al., 2019; Xiao et al., 2020). For the regulation of osteoclast activity, melatonin could suppress ROS-induced osteoclast differentiation and aging-mediated bone loss (Zhou et al., 2017; Tao et al., 2020). The nuclear factor Nrf2 plays an important role in oxidative-reductive signaling pathway-mediated bone remodeling (Han et al., 2022). Activation of Nrf2 not only maintains the survival of osteoblasts and mesenchymal stem cells but also inhibits osteoclast differentiation (Su et al., 2021; Wang et al., 2022b).

#### 2.4.2 Signaling pathway

Melatonin increases the expression of Nrf2 to promote osteogenic differentiation *via* the Wnt/β-catenin pathway and inhibits osteolysis by enhancing the activity of catalase (Vriend and Reiter, 2016; Zhu et al., 2020). Heme oxygenase 1 (HO-1) is an essential downstream target of Nrf2 in the process of regulating the oxidation-reduction response (Ma et al., 2019). HO-1 was regarded as the potential target for postmenopausal osteoporosis based on its physiological function of controlling carbon monoxide and ferritin iron to prevent cell apoptosis in bone tissue (Zhou et al., 2021). Melatonin is an agonist of the Nrf2/HO-1 signaling pathway (Yang et al., 2022b; Zhou et al., 2022). Melatonin also exerted antioxidant functions by activating the Nrf2/HO-1 signal to enhance intracellular antioxidant reactions (Guo et al., 2021b). In conclusion, melatonin plays an important role in the protection of bone metabolism from oxidative damage by enhancing the activity of antioxidase and scavenging free radicals *via* the Nrf2/HO-1 signaling pathway.

Targets and signaling pathways modulated by melatonin are listed in Table 3 to reveal the antioxidant effect of melatonin in bone balance.

**TABLE 3:** Targets and signaling pathways of melatonin exerting antioxidant effects in bone metabolism.

Targets and signaling pathways	Effect and function	Citation
SIRT1	Maintaining mitochondrial biosynthesis to improve oxidative damage	Liu et al. (2022b), Chen et al. (2020b)
SIRT3	Promoting antioxidase production to hydrolysis free radicals	Zhou et al. (2019), Xiao et al. (2020)
Nrf2	maintaining the survival of osteoblasts and mesenchymal stem cells, inhibiting osteoclast differentiation	Wang et al. (2022b), Su et al. (2021)
Nrf2/HO-1 signaling pathway	enhance intracellular antioxidant reaction	Guo et al. (2021b)

a Melatonin is the strongest endogenous free radical scavenger. SIRT1, Sirtuin 1; SIRT3, Sirtuin 3; Nrf2, NFE2 like bZIP, transcription factor 2; HO-1, Heme oxygenase 1.

### 2.5 Anti-inflammation and immunomodulation

#### 2.5.1 Pathogenesis

Estrogen has potential anti-inflammatory properties. Studies have demonstrated that estrogen receptor participates in the activation and proliferation of T lymphocytes, and estrogen also suppressed the production of proinflammatory cytokines by activating the NF-κB signaling pathway (Mohammad et al., 2018; Harding and Heaton, 2022). Therefore, postmenopausal osteoporosis has been regarded as a type of inflammatory disease (McLean, 2009). Immunological homeostasis also played an important role in the maintenance of bone balance. First, mononuclear macrophages are the precursors of osteoclasts. After combining with estrogen receptor α (ERα), estrogen inhibits the differentiation of mononuclear macrophages into osteoclast by inhibiting the secretion of IL-1β, TNF-α and IL-6 (Riggs, 2000). Estrogen could also reduce the secretion of IL-17 and RANKL, which promote osteoclast differentiation from CD4 T cells (Okamoto et al., 2017). Estrogen deficiency decreased the production of TGF-β, which could suppress osteoclasts with the inhibitory effect of IFNγ and TNF-α in T cells. Additionally, estrogen promoted the production of efficient B lymphopoiesis in bone marrow and reduced osteoclast differentiation by increasing OPG expression (Masuzawa et al., 1994; Fujiwara et al., 2016). The systemic immune-inflammation index is an important index for the prediction and diagnosis of bone mass disorders in postmenopausal women (Du et al., 2021).

#### 2.5.2 Molecular mechanism of anti-inflammation

Melatonin was revealed to inhibit the inflammatory response in recent studies (Xi et al., 2021; Zakria et al., 2021). Its anti-inflammatory mechanism mainly acts through the inhibition of inflammasome formation and the abolishment of proinflammatory factor expression (Liu et al., 2022c; Xiong et al., 2022). Melatonin promotes osteogenic differentiation *via* the Wnt/β-catenin pathway and suppresses the inhibitory effect of NF-κB on osteogenesis in an inflammatory environment (Li et al., 2019). Melatonin also overcame the IL-1β-induced weakening of the osteogenic capacity of mesenchymal stem cells and tumor necrosis factor-alpha (TNF-α) by increasing bone resorption of osteoclasts to improve inflammation-related osteoporosis (Liu et al., 2013; Lian et al., 2016).

### 2.5.3 Molecular mechanism of immunomodulation

Immune function is a necessary factor in the regulation of the inflammatory state of the body. Osteoimmunology is becoming the focus of research on bone metabolic diseases (Xu et al., 2021; Fischer and Haffner-Luntzer, 2022). Melatonin plays an important role in immune organs homeostasis and maturation of immunocytes. Melatonin regulated the immune network composition of hematopoietic lineage cells in bone marrow (Maestroni, 1998). The survival of T cells in the thymus and B cells in bone marrow depended on melatonin regulation (Yu et al., 2000). The immune response was also affected by melatonin. Melatonin combined with the membrane receptor MT1 in the spleen to increase immune parameters such as the spleen mass and lymphocyte and leukocyte counts (Vishwas and Haldar, 2013). The most direct effect of melatonin on immune function was to modulate the response of immune cells in peripheral lymph nodes, especially T cells (Álvarez-Sánchez et al., 2015). Melatonin inhibited the immune response of effective T cells and enhanced the function of regulatory T cells. As the precursors of osteoclasts, monocyte-derived macrophages are the direct link between the immune and skeletal systems (Yang and Liu, 2021). Melatonin inhibits the osteoclastogenesis and migration of macrophages to suppress inflammation-mediated bone resorption (Markus et al., 2021; Kim et al., 2022). Melatonin also acted on other immunocytes to modulate bone metabolism indirectly. First, melatonin inhibited the proliferation and differentiation of Th1 and Th17 cells, reducing the secretion of IFNγ, IL-17 and TNF-α to improve bone mass (Srivastava et al., 2018; Huang et al., 2022). Immature B cells were associated with bone mass by increasing the RANKL/OPG ratio, but melatonin could promote B cell activation (Luo et al., 2020; Titanji et al., 2020). The mechanisms of immune cells and inflammatory factors mentioned above are summarized in Table 4. Therefore, clarifying the role of melatonin in the modulation of the immune-bone link could improve the inflammatory state and bone mass loss in postmenopausal women with osteoporosis.

**TABLE 4:** Mechanism of melatonin-regulated immune cells and inflammatory factors involved in bone metabolism.

Immune cells	Inflammatory factors	Effect and mechanism in bone remodeling	Citation
T cells (Th1, Th17 and T regulatory cells)	IFNγ, IL-17 and TNF-α	Inhibiting the immune response of effective T cells and enhancing the function of T regulatory cells to suppress bone resorption	Álvarez-Sánchez et al. (2015), Huang et al. (2022), Srivastava et al. (2018)
B cells		Promoting B cell maturation to decrease RANKL/OPG ratio	Titanji et al. (2020), Luo et al. (2020)
Monocyte-derived macrophages		Suppressing inflammation-mediated bone resorption	Kim et al. (2022), Markus et al. (2021)
	IL-1β, TNF-α	Inhibiting inflammatory factors-induced weakening of the osteogenic capacity and increasing bone resorption	Lian et al. (2016), Liu et al. (2013)

a Neuroendocrine and immune systems are interconnected. IFNγ, interferon gamma; IL-17, Interleukin 17; TNF-α, tumour necrosis factor alpha; IL-1β, interleukin 1 beta; RANKL, TNF, superfamily member 11; OPG, TNF, receptor superfamily member 11b.

### 2.6 Gut-bone axis

#### 2.6.1 Pathogenesis

The “gut-bone axis” is a new concept that has been proposed in recent studies. If refers to the influence of the gut microbiota on the absorption of nutrients, which alters the blood metabolism factors involved in bone remodeling (Fernández-Murga et al., 2020; Gobron et al., 2020). The gut microbiota play an important role in bone homeostasis (Li et al., 2021b). Detection of the intestinal flora abundance in OVX mice indicated that the ratio of Firmicutes to Bacteroidetes was higher than that of normal female mice (Wen et al., 2020). Disorders in the composition of the gut microbiota led to changes in substance metabolism that resulted in bone imbalances (Greenbaum et al., 2022). In addition, the gut microbiota can also improve bone mass by promoting osteogenesis and inhibiting osteoclasts (Ding et al., 2020). Special microbiota such as Bifidobacterium longum could also enhance the immunomodulatory potential of regulatory B cells to improve bone mass (Sapra et al., 2022).

#### 2.6.2 Effect of melatonin on the gut

Melatonin is an essential factor in the regulation of the gut microenvironment. Melatonin primarily protects against gut barrier defects to control substance absorption and improves the microbiome to influence substance metabolism (Jing et al., 2022). In terms of substance absorption, melatonin altered intestinal permeability by regulating the expression of the intestinal tight junction proteins ZO-1, occludin and claudin-1 (Liu et al., 2022d). For substance metabolism, melatonin also had a positive effect on the diversity of the gut microbiome (Zhang et al., 2022). Additionally, melatonin protected the intestinal tract from pathological damage. Melatonin increased the activity of antioxidases, such as SOD, GSH-Px and CAT, and prevented the expression of oxidative damage-induced gastrointestinal apoptosis factors. (Khan et al., 2017). Melatonin also improved intestinal inflammation by increasing the production of anti-inflammatory cytokines (IL-10 and IFN-γ) and reducing the production of proinflammatory cytokines (IL-6 and TNF-α) (Wang et al., 2022c).

#### 2.6.3 Regulatory mechanism of melatonin on the “gut-bone axis”

It was found that melatonin could modulate the microbiota-dependent butyrate metabolism that prevents bone loss (Wu et al., 2021). Butyrate combined with its receptor GPR109A to alleviate osteolysis. In addition, increasing the absorption of Ca with the stimulation of melatonin directly contributes to the formation of the bone matrix (Areco et al., 2015). Melatonin improves the pathological state of the gut intestine by promoting Ca transportation through the transcellular and paracellular pathway (Diaz de Barboza et al., 2015). Melatonin adjusted gut metabolism and prevented gastric inflammation in OVX rats, which improved drug absorption for the treatment of osteoporosis (Gürler et al., 2019). Based on the evidence mentioned above, melatonin could improve the intestinal microenvironment, remodel the microbiota composition, regulate metabolism and absorption, and maintain the balance of bone tissue.

## 3 Discussion

Osteoporosis is characterized by decreases in bone quality and bone mass loss, which mainly occurs in postmenopausal osteoporosis (Lim et al., 2021). Estrogen deficiency weakens the inhibitory effects on osteoclasts by activating estrogen receptor (Cheng et al., 2022). Bone resorption of osteoclasts is enhanced beyond the bone formation ability of osteoclasts, resulting in the development of osteoporosis (Heubel and Nohe, 2021). To make matters worse, the onset of osteoporosis is insidious and it is usually detected when serious complications occur instead of during routine physical examination (Salamanna et al., 2015). Although many drugs have been designed for the treatment of osteoporosis that mainly targeting osteoclasts, their effect is not satisfactory. In addition, the bone mass of the patient is significantly reduced, and the bone quality has typically been significantly decreased when osteoporosis is diagnosed. Therefore, optimizing diagnostic criteria, improving treatment plans, and developing more effective drugs are of great benefit to the early diagnosis, prevention and treatment of osteoporosis.

Melatonin is an endocrine hormone that is secreted by the pineal gland. With its rhythmic secretion characteristics, melatonin regulates many physiological functions of the body. In recent years, researchers have paid more attention to the regulatory effects of melatonin on bone tissue. Neuropathological changes such as sleep disturbance and depression often appeared and were thought to be highly related to melatonin in women after amenorrhea (Yardimci et al., 2021). Previous studies have indicated that melatonin could act on GnRH neurons to inhibit estrogen synthesis by influencing the PKA, PKC, and MAPK pathways (Roy and Belsham, 2002). Melatonin is also regarded as a selective estrogen enzyme modulator (Gonzalez et al., 2008). Although serum melatonin levels appear to be inversely correlated with estrogen levels under physiological conditions, nocturnal melatonin levels were significantly lower in postmenopausal osteoporosis (Toffol et al., 2014). Additionally, serum melatonin levels also show a close relationship with osteogenic markers (Ostrowska et al., 2002). Imbalances in the circadian clock that are mediated by disturbances in melatonin secretion increase the risk of osteoporotic fractures in postmenopausal women (Feskanich et al., 2009). Therefore, serum melatonin measurements might become a new diagnostic standard for the prediction of the risk of postmenopausal osteoporosis. In fact, they could replace conventional dual energy X-rays, which would reduce economic costs and improve detection efficiency.

**FIGURE 2:**
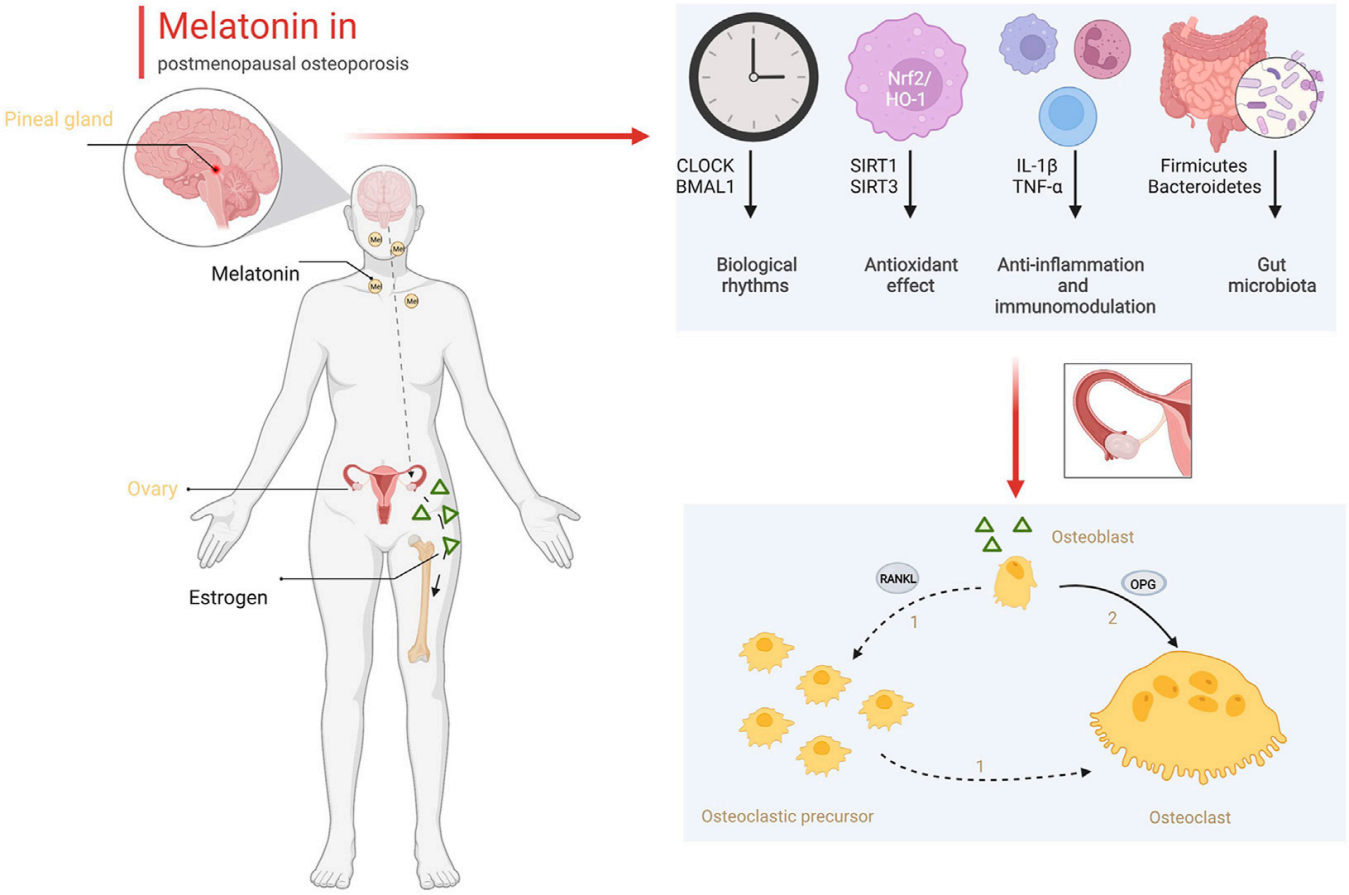
The role of melatonin in bone metabolism (Created with BioRender.com).

In addition to early diagnosis, melatonin also plays a potential role in the treatment of osteoporosis. Melatonin treatment positive affected the increase in bone mineral density and the improvement in body mass index (Treister-Goltzman and Peleg, 2021). However, oral estrogen could inhibit nocturnal melatonin release in postmenopausal women (Okatani et al., 2000). The therapeutic effect of melatonin mainly included a direct effect on bone and an improvement in the pathological state after estrogen deficiency, rather than improvement in estrogen levels. As we demonstrated in this review, melatonin directly promotes osteogenesis and inhibits osteoclastogenesis. Melatonin regulated the differentiation of BMSCs toward osteogenesis but not adipogenesis *via* the Wnt/β-catenin pathway and PPARγ pathway. In animal experiments, melatonin markedly improved bone mass in ovariectomized mice (Cao et al., 2022). Additionally, melatonin improves the microenvironment of bone tissue in postmenopausal women. As shown in Figure 2, estrogen synthesized by the ovaries intervenes in the expression of OPG and RANKL between osteoblasts and osteoclasts to maintain the balance of bone formation and bone resorption. Estrogen deficiency reduces the ratio of OPG/RANKL and promotes osteoclast proliferation and differentiation. Estrogen deficiency changes the daynight rhythm (Blattner and Mahoney, 2014). Biological rhythm disorder results in pathological states, including oxidative stress and an inflammatory storm (Sehirli et al., 2021; Yoshitane et al., 2022). Circadian disturbances mediate oxidative and inflammatory damage that lead to an imbalance in bone metabolism (Ostrowska et al., 2003; Oršolić and Jazvinšćak Jembrek, 2022). Melatonin could correct the circadian rhythm by regulating CLOCK and BMAL1, prevent oxidative stress by increasing the activity of antioxidant enzymes and reducing ROS production, and inhibit inflammation by reducing the secretion of inflammatory factors from immunocytes in bone tissue (Lee et al., 2018; Ren et al., 2018; Lu et al., 2021; Sadaf et al., 2021). Finally, melatonin remodels the gut ecology to promote the absorption of Ca and osteogenic substances, which also benefits the formation of the bone matrix and the strength of the bone structure. Based on these positive effects on bone metabolism, melatonin is an alternative drug that could be effective for the treatment of osteoporosis.

At present, more than one-third of postmenopausal women are at risk of osteoporosis and its complications, which seriously affect their life and health. Current diagnostic modalities and known treatments have limited effectiveness in improving the situation. The exploration of new diagnosis and treatment options is imminent. As an endogenous hormone, melatonin has been shown to be closely related to the occurrence and development of osteoporosis. In addition, melatonin has considerable prospects in the treatment of osteoporosis because of its limited side effects. Rational use of the advantages of melatonin will contribute to the diagnosis, prevention and treatment of postmenopausal osteoporosis.
